# Infant Feeding Practices That Substitute Exclusive Breastfeeding in a Semi-Rural Mexican Community: Types, Moments, and Associated Factors

**DOI:** 10.3390/nu14102017

**Published:** 2022-05-11

**Authors:** Erick Maas-Mendoza, Rodrigo Vega-Sánchez, Inocente Manuel Vázquez-Osorio, Solange Heller-Rouassant, María Eugenia Flores-Quijano

**Affiliations:** 1 Licenciatura de Nutrición, División Académica de Ciencias de la Salud, Universidad Juárez Autónoma de Tabasco, Villahermosa 86040, Tabasco, Mexico; maas24-93@hotmail.com (E.M.-M.); nutriologovazquez@outlook.es (I.M.V.-O.); 2 Departamento de Nutrición y Bioprogramación, Instituto Nacional de Perinatología Isidro Espinosa de los Reyes, Ciudad de México, Miguel Hidalgo 11000, Mexico; vegarodrig@gmail.com; 3 Jurisdicción Sanitaria 4 del Municipio de Centro, Secretaría de Salud, Villahermosa 86190, Tabasco, Mexico; 4 Academia Mexicana de Pediatría, Benito Juárez, Ciudad de México 03810, Mexico; solhrouas@gmail.com

**Keywords:** exclusive breastfeeding, breastfeeding, complementary feeding, infant feeding, breastmilk substitutes, formula milk, weaning, Mexico, Tabasco (Mexico)

## Abstract

International organizations recommend mothers practice exclusive breastfeeding (EBF) during the first six months of their infant’s life and introduce complementary feeding (CF) thereafter while continuing breastfeeding. However, the earlier introduction of liquids and foods is common worldwide and may have negative effects on breastfeeding practice, nutrition, and health. In this formative cross-sectional study, we interviewed 143 mothers from semi-rural communities in Tabasco, Mexico, whose infants were 4–6 months old. We explored (1) which feeding practices substituted EBF and (2) which factors were associated with each practice. During the first month of life, 42.7% of infants received formula milk (FM); this proportion increased to 74.5% by the sixth month. Adjusted Poisson regression analyses showed that giving FM was positively related to working away from home (PR 1.27; 95% CI 1.06, 1.54) and the perception that FM is an important food to accompany breast milk (PR 1.38; 95% CI 1.19, 1.70). Giving FM was negatively associated with not being sure the infant is full after breastfeeding (PR 0.75; 95% CI 0.61, 0.92). Regarding CF, less than half (47.5%) of infants had not received it by the fifth month. Factors positively associated with timely CF introduction were: the mother was told during prenatal care visits the optimal age to start CF is 6 months (PR 1.17, 95% CI 1.06, 1.29); she is convinced that giving only breast milk is best for her baby (PR 1.15, 95% CI 1.03, 1.29), and a higher infant weight-for-length (PR 1.04, 95% CI 1.00, 1.08) and length for age (PR 1.04, 95% CI 1.00, 1.09) z-scores at the study visit; conversely, it was negatively associated to the idea that if the infant is not full, she/he should receive formula milk or some other food (PR 0.87, 95% CI 0.78, 0.96). In these communities, EBF is lost to the use of FM and early CF. The factors associated with these inadequate feeding practices are related to returning to work, information received during prenatal visits, and the mother’s beliefs and thoughts. This work will guide the design of an intervention on infant feeding practices for these communities and other similar ones.

## 1. Introduction

Both the World Health Organization and UNICEF recommend that infants are exclusively breastfed for the first six months of life, which means that no other foods or liquids, including water, are provided to them during that period. Thereafter, age-appropriate and safe complementary foods should be introduced into their diet, while continuing to breastfeed for up to 2 years or beyond, if the mother and child so desire [1]. Nonetheless, the early introduction of nutritious and non-nutritive liquids, such as formula milk (FM) and plain water, as well as semi-solid and solid foods, has been widely documented [2,3,4,5]. This is a public health problem due to its negative effects on breastfeeding practice, nutrition, and health status [2], in the short and potentially long term.

The problem with the introduction and regular administration of FM or any other liquid or solid food is that it reduces breastfeeding frequency as well as sucking time and possibly thoroughness of milk removal from the breast. Eventually, this cascade of events triggers negative effects on breastfeeding, ranging from pain and discomfort in breasts and nipples to reduction in milk volume and early cessation of breastfeeding [2,6].

The potentially harmful effects of feeding FM on infant health have different forms. Some studies have documented that very early supplementation with cow milk-based formula to infants from susceptible families may sensitize them to develop cow milk protein allergy [7,8]. Exclusive FM and mixed milk feeding have been associated with the development of type 1 diabetes [9] and the alteration of the neonatal gut microbiome [10]. This shift in the gut bacterial profile may predispose the infant to infections, inflammation, and allergies and, possibly in the long term, to disease and conditions associated with dysfunction of the intestinal barrier such as inflammatory bowel disease and celiac disease [11].

Other adverse effects of inappropriate early infant feeding practices are, on one hand, an increased risk for growth faltering and micronutrient deficiencies which are commonly the result of two predisposing factors: (a) recurrent infections due to the use of contaminated liquids, solid foods, and infant feeding utensils [12,13]; and (b) insufficient energy and nutrient intake, from low nutritional value foods that replace breastmilk [14]. On the other hand, an accelerated weight gain may be prompted by the use of FM [15,16] and/or the early introduction of excessive and high energy content liquid and solid foods [17]. In the mid and long term, both deviations from what is considered normal growth may be detrimental to health with consequences such as short final stature, less physical and intellectual capacity, obesity, and chronic diseases [18].

In Mexico, the latest nationwide data (2018) show that only 25.2% and 37.4% of children under six months, in urban and rural communities, respectively, receive exclusive breastfeeding (EBF) [19]. Other surveys and studies have documented the consumption of FM, water, and other liquids [3,20] as well as the early introduction of complementary feeding (CF) [21] among infants younger than six months. 

The optimal and recommended way to carry out infant feeding has been extensively studied, and guidelines and programs have been proposed. However, the proven efficacy of such programs sometimes fails to translate into effectiveness in different contexts [22]. What happens in practice varies enormously depending on the geographical, cultural, economic, social, and even community or family contexts [23].

In the present community-based study, we aimed to describe: (1) which alternative feeding practices (e.g., FM use and early introduction of CF) substitute EBF; and (2) which sociodemographic, infant, and maternal factors are associated with each type of alternative breastfeeding practice. Factors were selected based on previous research [5,24,25]. The higher aim is to translate this knowledge and awareness into more effective strategies to promote infant feeding practices that are compatible with the greater health and well-being of infants and their families in the short and long term.

## 2. Materials and Methods 

### 2.1. Study Setting and Design

This is a secondary analysis of a formative cross-sectional research carried out in semi-rural communities from Tabasco, which is a coastal southeastern state in Mexico. A detailed description of the study setting and demographic characteristics can be found in a recently published paper [26]. Briefly, we included women beneficiaries of government social security programs who attended public health services and received prenatal care at the Health Center with Expanded Services (CESSA, initials in Spanish) located in the urban town of Villa Luis Gil Pérez or at one of the seventeen smaller first level public health units (FLPHU) located in rural or semi-rural villages, affiliated to CESSA. The study was approved by the Research and Ethics Committees of the National Institute of Perinatology (INPer) in Mexico City (212250-3310-11406-03-16) and authorized by the local health authorities at Centro Health Jurisdiction 04 in Tabasco. Data were collected from March to June 2016. 

We invited women to participate if they: (1) lived within the geographical limits of Villa Luis Gil Pérez or one of the other seventeen communities, (2) received prenatal care at CESSA or one of the FLPHU, (3) had a single and clinically healthy pregnancy, (4) had not been hospitalized for any condition that could be a barrier for breastfeeding initiation; (5) their infants were between 4 and 6 months old at the time of the study, and (6) accepted to participate and signed informed consent.

In the original study about exclusive breastfeeding [26], the sample size was calculated to estimate the proportion of women that would be breastfeeding exclusively, with 5% precision, 95% confidence level, and prevalence of exclusive breastfeeding in children under 6 months of 15.5%, which corresponded to the reported prevalence in the southern states of Mexico at the time [24]. With these parameters, the sample size was estimated to be 190–200 women. However, since we found that exclusive breastfeeding was much lower in the community, the target sample size was modified to 150 women.

### 2.2. Study Variables and Statistical Analyses 

To construct the study variables, we used items from an ad hoc questionnaire and some previously validated scales, which were applied by the researchers during the study visit. The questionnaire can be found in the Supplementary Material. The ad hoc questionnaire, including breastfeeding and complementary feeding practices, was developed specifically for this study by one of the authors and then revised by an expert pediatrician. The questionnaire was pilot tested with women from the community to verify that the questions and terms were properly understood. When questions were ambiguous or not correctly understood, we changed their composition and tested them again. In the study, we used the questionnaire that resulted after testing and revision. 

To describe the age at which different liquid and solid foods were first consumed by the infants, we asked: the infant’s age in months, the first time she/he received FM and other liquids (water, tea, juice, pozol, oatmeal with cocoa, atole, other) as well as the first time they received CF (vegetables, fruit, cereals and tubers, beans, meat, egg, dairy products). 

From these questions, we constructed two distinct outcome variables: (1) consumption of FM (yes/no) and (2) early or timely complementary feeding (ECF/TCF). The ECF group included women who gave CF at four months or earlier, while the TCF group included those who did it timely, i.e., from the beginning of the fifth month or later [27]. This last group also included women who had not started CF at the study visit (regardless of the infant’s age). 

Potential influencing factors (independent variables) were selected based on previous research [5,24,25], and these are described below.

#### 2.2.1. Sociodemographic Factors

Maternal sociodemographic factors included age (years), schooling (number of completed years of school), living with the infant’s father (yes/no), family type (uniparental/nuclear/extended), number of live births (no previous liveborn neonate/at least one previous liveborn neonate), and occupation (stays at home/works away from home). 

For estimating household socioeconomic level, we used the AMAI 8 × 7 Socioeconomic Level (SEL) Index, which was a questionnaire developed by the Mexican Association of Market Intelligence and Public Opinion Agencies (AMAI). It is based on a statistical model that classifies Mexican households into seven levels, according to the head of household’s ability to satisfy its members’ needs [28]. According to our study sample distribution, we recategorized the seven levels into two groups: high SEL (scores A/B, C+, C and C−) and low SEL (scores D+, D and E).

To estimate household food security, we used the Latin American and Caribbean Food Security Scale (ELCSA) adapted for the Mexican population. It consists of 15 questions with answer options “yes” or “no” addressed to the head of the family or to the woman in charge of preparing food at home. It enquires about experiences of hunger by household members and concerns about lack of food security, referring to the three months prior to the interview. Depending on the number of affirmative responses to each question, the ELCSA classifies households into four categories: (1) food security, (2) mild food insecurity, (3) moderate food insecurity, and (4) severe food insecurity [29].

#### 2.2.2. Maternal and Infant Factors

We considered mode of delivery (vaginal/cesarean section) and maternal cigarette and alcohol use at the time of the study visit (yes/no). Maternal weight and length were measured in triplicate at the study visit with equipment available at the CESSA or FLPHU using standardized protocols [30]. The mean for each anthropometric indicator was used to calculate maternal body mass index (BMI) (kg/height in square meters).

At the study visit, infants’ weight and length were measured using a Tanita baby and mommy scale model 1582 and a portable SECA infantometer model 207. Infant’s length-for-age (LAZ) and weight-for-length (WLZ) z-scores were calculated using the WHO Child Growth Standards with the Anthro Survey Analyzer [31].

#### 2.2.3. Previous Infant Feeding Experience, Breastfeeding Information, Difficulties, Beliefs, and Thoughts

We asked several questions to learn about maternal previous and present breastfeeding and CF experience beliefs and thoughts.

We constructed the categorical variable “previous EBF experience” based on our previous observation that early EBF abandonment is common in this population, with almost 60% of women having abandoned it by the first month [26]. Therefore, we grouped women who had never breastfed and those who practiced EBF with a previous infant for less than one month as “no previous EBF experience”. Women who had exclusively breastfed her previous child for two or more months were considered “with previous EBF experience”. 

According to WHO/UNICEF, all forms of feeding breast milk count as breastfeeding [32]; therefore, when referring to EBF in our study, we did not distinguish whether infants were sucking milk from their own mother’s breast or from a wet nurse or receiving expressed breast milk from their own mother or a donor.

Other categorical yes/no maternal factors considered were: not EBF at hospital discharge; the mother became sick and stopped breastfeeding; pain or discomfort on her breasts after hospital discharge; received free FM, bottles, or pacifiers during their prenatal visits or hospital; was told during their prenatal care visits that the optimal duration of EBF is six months and that CF should start at that age; and whether or not she received BF information or support during postpartum visits to the CESSA or FLHU.

To obtain some insight into what women believe and think about some aspects of infant feeding, and the influence these beliefs and thoughts had on the time they introduced CF, we asked them whether they agreed with the following statements:

- “Formula milk is an important food to accompany breast milk before six months”.
- “I am convinced that giving only breast milk until the baby is six months old, without giving any other food, is the best for her/him”.
- “When the baby is not full, you should give her/him powdered milk or some other food, even if she/he is less than six months old”.
- “If the baby is ‘small in size’ she/he should receive other foods to grow faster”.
- “When breastfeeding, you are always sure that your baby under six months is full”.
- “When it is very hot you should give your baby under six months water or some other liquid to drink”.

Mothers could respond on a Likert scale with five options ranging from “strongly agree” to “strongly disagree”, and a sixth option: “don’t know”. These six options were then narrowed down to two categories: “agree” and “disagree”, which were used in the analyses. 

#### 2.2.4. Statistical Analyses

We first performed bivariate analysis to establish the association between the outcome variables and each independent variable. To select the variables for the models, we used Chi-square, Student’s-*t*, or Mann–Whitney’s U test depending on variable type and distribution. Independent variables that were at least marginally (*p* ≤ 0.10) associated with the outcome variable were included in a Poisson regression computed through a General Linear Model considering a log-link function and a robust covariance estimator. We checked the uptake of variables for collinearity and accepted correlations <0.40; tolerance > 0.75; and FIV < 1.5.

All statistical analyses were carried out in SPSS version 21.

## 3. Results

### 3.1. Study Population

We invited 200 women to participate; 28.5% of them did not meet the inclusion criteria because they did not receive their prenatal care at CESSA (*n* = 24), their babies were older than 6 months (*n* = 22), presented complications during birth (*n* = 8) or did not accept to participate (*n* = 3). Therefore, a total of 143 women were included in the final sample.

Table 1 shows the general characteristics of the 143 mother–infant dyads included in the final sample. None of the study participants smoked, and only a few (2.1%, *n* = 3) drank alcohol around the time of the study visit.

Most households belonged to the low SEL category. According to AMAI classification, such households range from a total lack of space and sanitary infrastructure to barely covering them. They also allocate a significant proportion of their income (between 42 and 52%) on food, leaving other needs uncovered.

*Previous Infant Feeding Experience, Breastfeeding Information, Difficulties, and Thoughts*

Women in the “no previous EBF experience” group comprised 68.5% (*n* = 98) of the study sample. Most women with at least one previous live birth had breastfed their previous child (83.7%, *n* = 72), but only around half of them had practiced EBF beyond the first month of life (52.3%, *n* = 45). 

We asked women if during their prenatal care visits, they had received information on EBF and the introduction of CF. More than half (60.8%, *n* = 87) said they received information on EBF duration and 45.5% (*n* = 65) were told the optimal breastfeeding duration is 6 months. Regarding CF, 43.4% (*n* = 62) reported receiving information about the time for introducing CF but only in 32.9% of cases (*n* = 47) was such information correct (i.e., TCF). In addition to the information received at health centers, women also reported receiving information on infant feeding from the television (50.0%), magazines (44.1%), radio (23.1%), newspapers (23.1%), and other sources (24.5%, e.g., social networks). However, we did not inquire about the type or quality of such information.

After giving birth, most women left the hospital practicing EBF (75.5%, *n* = 108); only 2.8% (*n* = 4) reported receiving free formula, bottles, or pacifiers during their prenatal visits or hospital stay. During the following weeks, 14% (*n* = 20) of women said they suffered a disease that forced them to stop breastfeeding, although most were able to resume breastfeeding again (*n* = 14). Around half of our study sample (49.7%, *n* = 71) experienced pain or discomfort in breasts and/or nipples, but only one-fifth (19.6%, *n* = 28) received support or breastfeeding information at the CESSA or FLHU.

An important proportion of women in our sample had beliefs or thoughts that may increase the risk of introducing FM and ECF. Around half of the mothers agreed that “Formula milk is an important food…” (50.3%, *n* = 72) and “When the baby is not full, you should give powdered milk…” (53.1%, *n* = 76). One-third of them disagreed with the ideas “I am convinced that giving only breast milk… is best for her/him” (32.9%, *n* = 47) and “When you finish breastfeeding… your baby is full” (35%, *n* = 50). Some agreed that “If the baby is small should receive other foods…” (34.3%, *n* = 49).

### 3.2. Consumption of Liquids

**Figure 1:**
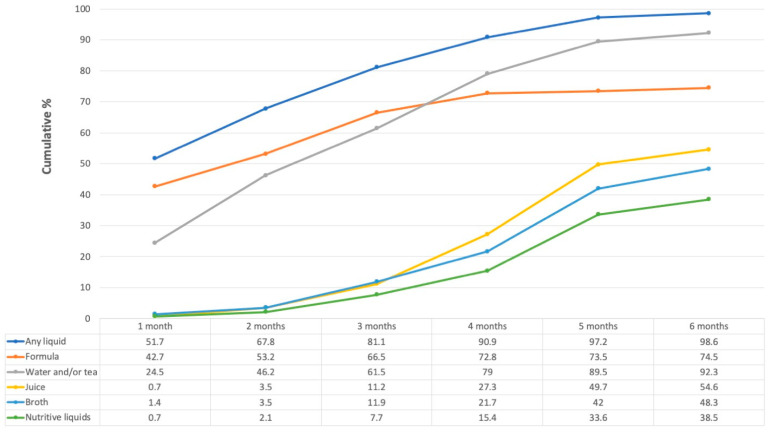
Liquid foods consumed over the first six months of life. Numbers in the table represent percentages. The “Any liquid” category is not the sum of the other categories, since some women could have given more than one liquid during the same month. “Broths” could be made of beans, chicken, beef, fish, lentils, or vegetables. “Nutritive liquids” include atole (maize flour with water/milk), pozol (maize dough with water and with or without cocoa), and oatmeal with cocoa.

Figure 1 shows the most consumed liquids in the first six months of life. Nearly half of infants (42.7%, *n* = 61) received FM during the first month, 66.5% (*n* = 95) by the third month, and the proportion reached 74.5% (*n* = 107) by the sixth month.

Water and tea were also given to almost one-quarter (*n* = 35) of the infants during the first month, the proportion rising to 61.5% (*n* = 88) at three months and continuing to increase to 92.2% (*n* = 132) by six months. Other liquids such as juice, broth, and nutritive liquids were given to a small percentage of infants during the first three months, their consumption increasing at a slower pace. At six months of life, these liquids were given to around 40 to 55% of infants. 

Since the climate in Tabasco is very hot most of the year, it is common to hear those infants receive water and tea to keep them hydrated. Most of the participants (76.9%, *n* = 110) agreed with the sentence: “When it is very hot you should give your baby under six months water or some other liquid to drink”.

*Which Women Gave Formula Milk?*

We wanted to understand the factors associated with the use of FM, since it is the earliest and most frequently given liquid food to infants. We compared women who had given FM at any given point of their infant’s life (formula group, 74.6%, *n* = 106) to those who had never given it (no formula group, 25.4%, *n* = 36). More women in the formula group had at least one previous live birth (46.7%, *n* = 50 vs. 19.4%, *n* = 7; *p* < 0.01), worked outside their home (14%, *n* = 15 vs. 2.8%, *n* = 1; *p* = 0.07), and were not living with their infant’s father (16.8%, *n* = 18 vs. 5.5%, *n* = 2; *p* = 0.09). Maternal BMI, mode of delivery, infant’s sex, LAZ, WLZ, and the sources of information about infant feeding were not different between groups.

Regarding previous infant feeding practices, more women in the formula group had never breastfed or, if they did, they had practiced EBF for less than one month (75.7%, *n* = 81 vs. 47.2% *n* = 17; *p* < 0.01). These women were told during their prenatal care visits to practice EBF during the first six months of their infant’s life (49.5%, *n* = 53 vs. 33.3%, *n* = 12; *p* = 0.09). There was no difference between groups in any other study variable. 

Regarding the participants’ thoughts about FM and breastfeeding, more women who gave formula agreed with the phrases: “Formula milk is an important food…” (59.8%, *n* = 64 vs. 22.2%, *n* = 8; *p* < 0.01) and “When the baby is not full, you should give powdered milk…” (57.9%, *n* = 62 vs. 38.9%, *n* = 14; *p* = 0.02). In contrast, more women who had not given FM agreed that “When you finish breastfeeding… your baby is full” (86.1%, *n* = 31 vs. 57.5%; *p* < 0.01) and “I am convinced that giving only breast milk… is best for her/him” (83.3%, *n* = 39 vs. 61.7%, *n* = 66; 0.06) 

In a model adjusted by all significant variables (Table 2), women were more likely to give FM if they worked away from home or agreed with the idea that FM is an important food; and they were less likely to do so if they were sure their infant is full after breastfeeding.

### 3.3. Consumption of Solid Foods 

At the time of the study visit, 89.9% (*n* = 120) of infants had initiated CF. All infants were 6 months old or younger.

**Figure 2:**
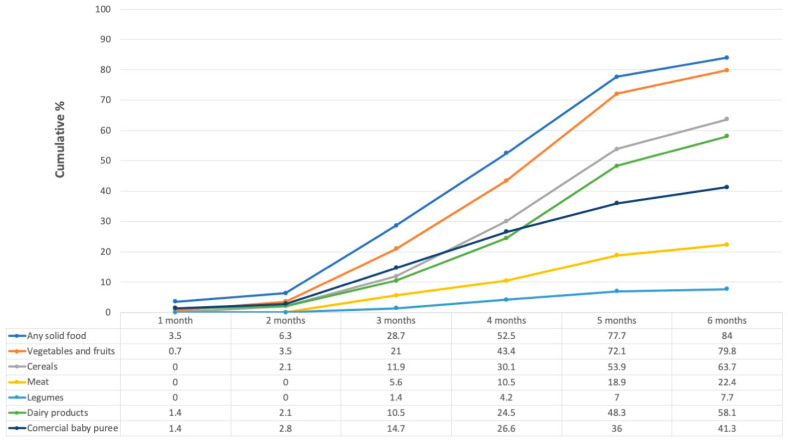
Cumulative percentage of solid foods consumed over the first six months of life. Numbers in the table represent percentages. Those in the “Any solid food” category are not the sum of the other categories, since some women could have given more than one food during the same month.

As Figure 2 shows, few infants were given CF during their first (3.5%, *n* = 5) or second (2.8%, *n* = 4) months of life. Thereafter, the proportion increased, and by the fourth month, more than half (52.5%, *n* = 75) had had some solid food. Fresh vegetables, fruits, and cereals were the most given solid foods, which was followed by dairy products (petit-Suisse cheese and yogurt), commercial baby puree, and, to a lesser extent, meat, and legumes.

We asked women who had begun CF (89.9%, *n* = 120) about their reasons for doing so. The most common responses were: “the baby was hungry” (36.4%, *n* = 52), “the baby craved for food” (12.6%, *n* = 18), “the baby was ready/was old enough to receive them” (11.9%, *n* = 17), “a friend or family member recommended it’’ (9.8%, *n* = 14) and “the doctor recommended it’’ (7.7%, *n* = 11). Most mothers who responded “the baby was ready/was old enough to receive them” were in the timely complementary feeding (TCF group) (24.4%, *n* = 11 vs. 8%, *n* = 6; *p* = 0.01); no other answer showed a difference between early complementary feeding (ECF) and TCF groups.

*Which Women Started Giving Complementary Food before the Fifth Month?*

Women were almost evenly distributed between the ECF (52.4%, *n* = 75) and TCF (47.6%, *n* = 68) groups. Women in the ECF group were marginally younger than their counterparts in the TCF group (mean 23.13 ± 5.93 vs. 24.94 ± 6.38; *p* = 0.08). Other sociodemographic characteristics did not differ between groups. 

In the TCF group, there was a higher proportion of women who had been advised to start CF at six months during their prenatal care visits (41.2%, *n* = 28 vs. 25.3%, *n* = 19; *p* = 0.04) and tended to have EBF their previous infant for at least one month (38.2%, *n* = 26 vs. 25.3%, *n* = 19; *p* = 0.09). The groups were not different in the proportion of women who became sick and stopped breastfeeding, experienced pain, or discomfort in breasts and/or nipples, gave FM to their infant or received breastfeeding information or support during postpartum.

Regarding the influence of maternal beliefs and thoughts about the introduction of CF, more women in the TCF group agreed with the thought “I am convinced that giving only breast milk… is best for her/him” (77.9%, *n* = 53 vs. 57.3%, *n* = 43; *p* = 0.03). In contrast, more women in the ECF group agreed with “When the baby is not full, you should give powdered milk…” (64%, *n* = 48 vs. 41.2%, *n* = 28; *p* = 0.02) and “If the baby is small, she/he should receive other foods…” (41.3%, *n* = 31 vs. 26.5%, *n* = 18; *p* = 0.02). 

There was no difference in the proportion of girls and boys between ECF and TCF groups. Infants in the TCF group had higher mean LAZ (−0.192 ± 1.34 vs. −0.806 ± 1.19; *p* < 0.01) and WLZ (0.525 ± 1.25 vs. 0.122 ± 1.20; *p* = 0.05). Women in the TCF group had higher BMI at study visit (27.44 ± 5.33 vs. 25.50 ± 5.50; *p* = 0.03). 

A Poisson regression model showed that the strongest factors related to starting CF beyond the fourth month (Table 3) were: if women received prenatal information about the optimal time to start CF and her thoughts about infant feeding. The infant’s size (weight-for-length and length-for-age) was marginally significant.

## 4. Discussion

This research provides a detailed view of infant feeding practices during the first six months of life in a semi-rural Mexican community. Women give up breastfeeding prematurely and instead supplement mainly with FM; around 43% gave it during the first month, but by the end of the first semester, three-quarters of the mothers were feeding FM to their infants. Non-nutritive liquids such as water and tea also displace EBF, almost one-quarter of the women giving them as early as the first month and nearly all of them (92%) by the sixth month. Almost one-third of the women began CF around the third month of age by giving their infants solid food for the first time.

### 4.1. Early Introduction of Liquids

#### 4.1.1. Formula Milk 

In Mexico, FM is most frequently introduced as the first liquid food into the infants’ diet, as has been described by national surveys and previous studies. Data from the ENSANUT (National Health and Nutrition Survey) 2012 included 1015 infants younger than 6 months, of which 56.7% received FM; 29% were also breastfed, and 26.69% no longer received human milk [3]. In the most recent national survey (ENSANUT 2018–2019), 25.6% of infants younger than 6 months received mixed milk feeding, albeit the total number of infants receiving FM is not reported [33].

More detailed information indicates that FM introduction during the first month is common, and figures very close to the 43% we observed in this study have been reported both in national surveys [34,35] and in a smaller longitudinal study conducted in the states of Chihuahua and Puebla. In this last study, the authors reported that overall, 47.7% of women had introduced FM during the first month. However, this proportion was different among those who attended public vs. private hospitals (44.3% vs. 74.4%, *p* < 0.01) and those who lived in rural vs. urban areas (32.4 vs. 56.6%, *p* < 0.001) [36]. Other studies have also reported that by the time infants are 6 months old, a large proportion of them (such as 70%) are receiving FM [34].

We identified some maternal sociodemographic characteristics and thoughts that promote or discourage FM use in our study population. Infants are more likely to receive FM if the mother works outside her home, agrees that “Formula milk is an important food to accompany breast milk before six months”, or disagrees with the thought “When you finish breastfeeding, you are always sure that your baby under six months is full”. These findings are consistent with the evidence from studies in Mexico and other low- and middle-income countries (LMIC). Working outside the home has been linked to the reduction in EBF [37,38,39] by introducing FM [21,40,41,42]. For example, in Bangladesh, most mothers with formal employment in ready-made garment factories introduced FM as early as two months, despite knowing that breast milk is the sole ideal food for infants [41].

However, it has been previously described that it is not the maternal place of work, but rather specific characteristics of employment, that influence infant feeding practices. Some employment conditions related to FM use are separation of the infant’s mother, non-flexible shifts, difficult transportation conditions, lack of childcare, and/or an exclusive and properly equipped breastfeeding room where women may breastfeed or express and store milk, and maternity leave policies [40,43]. To propose specific interventions for working mothers in this or other communities, it would be necessary to obtain more detailed information regarding their employment conditions.

The belief that FM is an important food to accompany breastfeeding (i.e., a complement to breastfeeding) has been documented in previous studies, showing the belief’s role as a barrier to EBF and its association with the introduction of FM to the infant’s diet [36]. Studies in Mexico have described that although the majority of mothers agreed to give EBF during the first six months because they thought it is best for their infants, in practice, they exercised mixed milk feeding [21]. The data suggested that women did not differentiate between breastfeeding and EBF [21,36]. This means that possibly, women are unaware of the potential harm and disadvantages of FM feeding, while believing that both types of milk can be used interchangeably to comply with EBF recommendations. Indeed, this seemed to be the case with some of our study’s participants, as we learned from their informal comments (not directly asked as part of our questionnaire). This belief could partly explain why women opt to introduce FM when they face difficult circumstances, such as perceiving insufficient milk supply, separation from their infant, or having sore or cracked nipples [36].

In Indonesia, many caregivers also believe that FM feeding and breastfeeding are equally good options to feed their infants, and mixed milk feeding in infants under 6 months is widespread. A study documented that for participant mothers, the most important motivators for FM use were the perceived benefits on child growth, intelligence, and immunity. The study also revealed that there was almost universal exposure to the marketing of breast milk substitutes. The authors proposed that health and nutrition claims from FM vendors may influence caregivers and their feeding choices [42].

Regarding exposure to FM marketing in our study, we only asked women if they had received free FM, bottles, or pacifiers during their prenatal visits or hospital stay. Very few (3%) women answered affirmatively. However, we did not explore their exposure to inappropriate marketing strategies outside the health system, either through publicity in mass media, points of sale, or the presence of nutritional and health claims on the products’ labels. These are the places where most of the FM promotion has been observed in Mexico and other countries [42,44].

The mother’s perception that the infant is still hungry after breastfeeding, suggesting there is insufficient milk production to make her infant feel full, has been consistently recognized as a reason for feeding FM [21,44,45,46]. We also documented this belief in our study and found it to be a factor significantly associated with the practice of giving FM. Since only a very small number of women are physically unable to produce an adequate volume of milk for their infant [47], the perception of this inadequacy is rather the result of a lack of knowledge and understanding of the normal physiological process of lactation or unrealistic expectations of breastfed infant behavior. A newborn’s frequent need to feed due to their small stomach capacity [48] or their crying [46] are behaviors often wrongly interpreted as hunger. Crying, in fact, is a late sign of hunger and comes after other feeding cues; it is also a signal to many other needs such as comfort, warmth, maternal presence, or pain, illness, or fear [48].

Regarding lactation physiology, most women experience normal lactogenesis and have the potential to produce an abundant milk supply for their infants from the start. Nevertheless, inadequate breastfeeding techniques, practices, or difficulties interfere with constant and efficient milk removal from the breast. This soon leads to diminished milk production. Factors that have been shown to interfere with milk removal are scheduled feeds, the introduction of FM or other liquids (without the corresponding expression of milk), and the use of pacifiers that reduce the time of breast sucking, among others [47,48]. The design of an intervention to promote EBF in the community should include a strategy to reinforce the caregivers to recognize normal or expected feeding and sleeping patterns to correctly respond to their infant’s needs.

Other factors such as socioeconomic level, maternal schooling, area of residence (urban vs. rural), type of health unit (private or public), and age group (adolescents vs. adults) have also been identified to influence feeding FM [36,42]. However, our study population was quite homogeneous in these factors, since all lived in semi-rural areas and mostly gave birth in the same public hospital.

#### 4.1.2. Water and Other Non-Nutritive Liquids

The early introduction to the infant’s diet of water, tea, or other non-nutritive liquids has been previously observed in Mexico and other LMICs. In Mexico, a national survey in 2012 documented that 51% of infants younger than 6 months received water and 25.7% received water-based drinks (tea, broth, coffee, soft drinks, and juices) [3]. A smaller study held in the Mexican states of Querétaro and Oaxaca also documented that giving water was a common practice, starting around two months after birth [21,49]. In other countries, such as Guatemala [49] and Senegal [50], studies show that a large proportion of infants younger than six months (79% and 85%, respectively) are introduced to water or tea, which are figures close to what we observed in Tabasco (89.5%). The most prevalent reasons to offer these beverages to infants are: (1) to quench thirst [21,41], (2) to alleviate gastrointestinal complaints, fever or to relax the baby [21,49], and (3) as a ritual with some cultural meaning [49,50]. Another possible reason is the fact that some women may not distinguish the difference between EBF and non-EBF, as we mentioned earlier for FM. This was also described in the above-mentioned study of Mexican women from Querétaro and Oaxaca, who answered that EBF also includes water and tea [21].

In our study setting, where the climate is very hot, women usually give water to their infants, believing it will relieve their thirst. However, since breast milk is at least 80% water, especially the milk that first comes at each feed, even in hot climates, breast milk is the best liquid to satisfy the infant’s thirst [51]. Additionally, a previous nutrition survey in Tabasco reported that children younger than 1 year with acute diarrhea (AD) were treated with water (plain or fruited, 56.2%), tea or atole (5.6%) or other liquids (17.6%); in addition, 17.5% of cases were given milk to treat diarrhea, but it was not reported whether it was breastmilk or FM [52].

We did not explore if water or the other non-nutritive liquids were given for other reasons different than those already discussed. In order to develop an intervention with an assertive and respectful message for discouraging the early introduction of water and water-based beverages to infants, it is necessary that we investigate this topic further.

### 4.2. Early or Timely Introduction of Complementary Feeding

As with the use of FM and other liquids, the early introduction of CF is a common practice in Mexico and other countries. A study in the Mexican state of Hidalgo reported that a large proportion of infants (57%) started receiving CF at three months of age [14]. In Querétaro and Oaxaca, women reported giving their babies “probaditas” or little bites of food as early as 3 to 4 months [21]. In other LMICs, a large proportion of infants also receive CF earlier than recommended [25,50,53].

Our study showed that four factors were strongly associated with TCF: (1) the mother was told during prenatal care visits that the optimal age to start CF is 6 months; (2) the mother disagreed with the phrase “When the baby is not full, you should give powdered milk…”; (3) or agreed with the phrase “I am convinced that giving only breast milk… is best for her/him” and (4) higher infant size: LAZ and WLZ. It was foreseeable that the information about CF women received during their prenatal care visits would influence their practice, as had been previously documented in other low-resource communities in Mexico [21]. It is noticeable that a good number of women in our study responded that they did not receive information on CF or recalled erroneous information. This means that an important part of an infant feeding intervention at the community level would be to train health workers on TCF practices and make sure all women receive and understand such information.

Although we did not explicitly explore maternal breastfeeding self-efficacy (BFSE, the self-confidence of a mother in her ability to adequately feed her infant [54]), two beliefs closely related to BFSE were associated with TCF in our study. First, the woman’s belief that the infant “is full” after a feeding; and second, the maternal conviction that giving only breast milk is the best for her/him. As we discussed above, the first belief is related to the introduction of FM and may result from incorrect or unrealistic expectations of infant behavior, or from a lack of understanding of the physiology of lactation. However, the second belief is more likely the result of a complex interaction of factors, including the woman’s previous breastfeeding experience, the amount and type of information about TCF she received during pregnancy, and the type and magnitude of influence from her social network (family, peers), among others. Therefore, this complex interaction of perceptual, informational, and social factors must be considered to promote maternal self-efficacy when designing an infant feeding intervention at the community level.

With respect to infant size, in our study, smaller infants were prone to receive ECF. However, due to the cross-sectional design of the study, we were unable to distinguish between two possibilities that may explain this association: either infants who received ECF suffered weight faltering or smaller infants are more likely to receive ECF. In underdeveloped countries, it has been described, on one hand, that while infants grow steadily when breastfed exclusively, when CF is begun, they may suffer growth faltering as a result of infections and poor-quality liquid and solid foods. On the other hand, it has also been observed that in countries where undernutrition is common, ECF to small infants is considered a “good thing” to promote catch-up growth, survival, and better health [18].

From these two possibilities, we consider that the second explanation may be appropriate for our study population, since another associated factor with TCF was the mothers’ disagreement with the phrase “If the baby is ‘small in size’ she/he should receive other foods to grow faster”. A similar observation was previously documented in a study in the Gambia, where higher weight-for-length predicted the introduction of CF at an older age. The authors suggest that the influence of infant size on the initiation of CF may be due to the mothers’ choosing to continue EBF when their infant is growing well [55].

### 4.3. Study Limitations

There are some limitations to our study, such as its cross-sectional design, which entails a key disadvantage: the retrospective nature of data collection that raises a possible recall bias, specifically about the precise moment in which participants introduced CF to their infant’s diet. However, infants’ age at the time of the study was near the time of introduction, possibly reducing such bias.

The study sample was rather small, and due to the study’s objectives and design, it was also quite homogeneous. The latter would imply that most of our observations may not represent women living in other settings. However, together with data from national surveys and information reported from other regions of Mexico [17,21,35], our results contribute to the knowledge of infant feeding practices in the country and highlight factors that promote or prevent those which are beneficial to nutrition and health.

### 4.4. Conclusions

The use of FM, water, and the early introduction of complementary foods are common infant feeding practices that substitute EBF in the community we studied. We identified a number of factors associated with these practices. 

Not all women in the community receive support and information on infant feeding practices during prenatal and postpartum visits, and when they do, sometimes, such information is incorrect. This reveals the need to further study the infant feeding knowledge and beliefs health providers have, design a training strategy for them, and integrate counseling on infant feeding as a central part of women’s care.

Women in the community have beliefs that negatively influence their infant’s feeding practices, reflecting a lack of understanding about the physiology of breastfeeding and incorrect or unrealistic expectations about the behavior and growth of breastfed babies. Such beliefs include giving water and other non-nutritive liquids to relieve thirst in the hot climate or the idea that FM is an important food in an infant’s diet. The latter being so widespread an idea suggests that FM is being normalized and possibly considered a desirable practice. More qualitative research is needed to delve into this hypothesis and propose strategies to modify how FM is perceived in this population. Additionally, the mother working outside the home was the factor most strongly associated with the use of FM. It is important to deepen the research to know the type and conditions of maternal employment and be able to design an intervention that responds to the specific needs of working women in this community and makes breastfeeding feasible for them.

In general, we found that beliefs about infant feeding are the factors that ultimately influence the way infants are fed. It is important to design and implement strategies that respectfully modify beliefs and promote an environment that normalizes breastfeeding through the information available from health services and the media, among other influential sources. 

## Figures and Tables

**Table 1:** Maternal and infant characteristics.

Maternal Characteristics (*n* = 143)	
Maternal age (years, median, p25–p75)	23 (19–27)
Schooling (years, median, p25–p75)	9 (8–12)
Lived with their infant’s father (*n*, %)	123 (86.0)
Stay-at-home mother (*n*, %)	127 (88.8)
Family type nuclear household (*n*, %) extended family (*n*, %) monoparental (*n*, %)	79 (55.0) 60 (42.0) 4 (2.8)
At least one previous live birth (*n*, %)	86 (60.1)
Low socioeconomic level (lowest three AMAI SEL categories) (*n*, %)	105 (73.0)
Food insecurity mild (*n*, %) moderate (*n*, %) severe (*n*, %)	68 (47.6) 26 (18.2) 16 (1.2)
Maternal BMI * (median, p25–p75)	25.63 (22.6–29.0)
**Infant Characteristics** **(*n* = 143)**	
Vaginal delivery	113 (79%)
Infants’ sex	76 female (53.1%)
Infants’ age * (months, median, p25–p75)	5.75 (4.8–6.3)
Infants’ LAZ (median, p25–p75)	−0.67 (−1.33–0.35)
Infants’ WAZ (median, p25–p75)	−0.14 (−0.98–0.63)

* At study visit.

**Table 2:** Factors related to giving formula milk.

Factor	PR (Adjusted 95%CI)	*p*
Occupation		
Works away from home (yes)	1.27 (1.06, 1.54)	0.011
“Formula milk is an important food to accompany breast milk before six months”		
Agree (yes)	1.38 (1.19, 1.70)	0.003
“When you finish breastfeeding, you are always sure that your baby under six months is full”		
Agree (yes)	0.75 (0.61, 0.92)	0.007

We excluded the number of live births, since it was highly correlated with previous EBF experience. Predicted probabilities are for being in the “giving formula milk” group. PR, prevalence ratio. Variables not significant in the model: lived with the infant’s father; previous EBF experience, received EBF information (duration for six months) during pregnancy, “When the baby is not full, you should give powdered milk…” and “I am convinced that giving only breast milk… is best for her/him”.

**Table 3:** Factors related to starting timely complementary feeding *.

	PR (Adjusted 95%CI)	*p*
During her prenatal care visits, the mother was told the optimal age to start CF is 6 months.		
Yes	1.17 (1.06, 1.29)	0.002
“I am convinced that giving only breast milk until the baby is six months old, without giving any other food, is the best for her/him”		
Agree	1.15 (1.03, 1.29)	0.012
“When the baby is not full, you should give her/him powdered milk or some other food, even if she/he is less than six months old”		
Agree	0.87 (0.78, 0.96)	0.009
WLZ		
(at study visit)	1.04 (1.00, 1.08)	0.022
LAZ		
(at study visit)	1.04 (1.00, 1.09)	0.040

* Includes 20 mother–infant dyads who had not initiated CF at their study visit, although they were younger than 5 months. Predicted probabilities are for being in the TCF group. PR, prevalence ratio. Variables not significant in the model: maternal age and previous EBF experience, “If the baby is small, she/heshould receive other foods…”, maternal age and BMI.

## Data Availability

The raw data supporting the conclusions of this article will be made available by the authors without undue reservation.

## Supplementary Materials

The following supporting information can be downloaded at: https://www.mdpi.com/article/10.3390/nu14102017/s1, Supplementary Material. Breastfeeding and complementary feeding practices questionnaire.

## Abbreviations

AMAI	Spanish abbreviation for Mexican Association of Market Intelligence and Public Opinion Agencies
BFSE	breastfeeding self-efficacy
BMI	body mass index
CESSA	Spanish abbreviation for health center with expanded services
CF	complementary feeding
EBF	exclusive breastfeeding
ECF	early complementary feeding
ELCSA	Latin American and Caribbean Food Security Scale
ENSANUT	National Health and Nutrition Survey (Mexico)
FIV	variance inflation factor
FLPHU	first-level public health units
FM	formula milk
LAZ	length-for-age z-score
SEL	socioeconomic level
TCF	timely complementary feeding
WLZ	weight-for-age z-score
