# Supplementary material for: Infant Feeding Practices That Substitute Exclusive Breastfeeding in a Semi-Rural Mexican Community: Types, Moments, and Associated Factors
Source: Nutrients. 2022 May 11;14(10):2017. doi: 10.3390/nu14102017 (PMC9146950; doi:10.3390/nu14102017)
Supplement: Supplementary file 1 [file nutrients-14-02017-s001.zip › nutrients-1649331-supplementary.pdf]

Supplementary Material. Breastfeeding and complementary feeding practices questionnaire.

Section A: MOTHER'S ID AND SOCIODEMOGRAPHIC DATA

1. Name
2. Birth date
3. Age (years)
4. Address
5. Phone number
6. Do you live with your baby's father? ( ) yes ( ) no
7. What is your family type?

( ) Monoparental (you live alone with your bay and other children)
( ) Nuclear (You and your baby's father live together with you children)
( ) Extended with the mother's family (you and your partner live with your family)
( ) Extended with the father's family (you and your partner live with your partner's family)

8. What is your last completed school grade?
9. What is your current occupation?

( ) Student
( ) Housewife
( ) Employee, profesionist o businesswoman
( ) Other

10. Do you currently use any of these substances?

( ) yes ( ) no Cigarettes, how many a day?
( ) yes ( ) no Alcohol, times per week?
( ) yes ( ) no Other (specify)

Section B: LAST PREGNANCY

1. Is this your first baby? ( ) yes ( ) no

(If yes, go to question 4)

2. How many pregnancies have you had? (including this baby)
3. How many of you babies were born alive?
4. During your last pregnancy, did you attend CESSA Villa Luis Gil Pérez or any of its medical units for prenatal care? ( ) yes ( ) no, where?
5. Type of birth

( ) Vaginal
( ) Cesarean section
( ) Elective Cesarean section
( ) Emergency procedure
( ) Don't know

6. When was your last menstrual period? Date / Don't remember
7. When was your baby's birthdate? day/month/year
8. How many gestational weeks did your baby have at birth? Weeks / Don't know
9. What is your baby's sex?
10. What was your baby's birth weight? Kg / Don't remember
11. What was your baby's birth length? Cm / Don't remember

Section C: PREVIOUS BREASTFEEDING EXPERIENCE
If this is the woman's first baby, go to the next section.

1. What is the birthdate of your previous child? day/month/year
2. How old is your previous child today? Years / Months
3. Did you ever breastfeed your previous child? ( ) Yes ( ) No
If Yes, go to question 7.
4. If not, for what reason(s) did you not breastfeed her/him?
5. How old was your previous child when you stopped breastfeeding her/him exclusively. In other words, when did you give her/him any food other than breastmilk (including water, formula milk, tea, cereals, juices or other food)? Months / Weeks / Don't remember
6. How old was your previous child when you last breastfed her/him? In other words, the last time you nursed her/him. Years / Months / Weeks / Don't remember

Section D: BREASTFEEDING INFORMATION
1. During your prenatal care at CESSA or medical unit, did you receive information regarding the following subjects?
a) That you should exclusively breastfeed your baby. ( ) Yes ( ) No / For how long?
b) The optimal age for offering your baby food other than breastmilk.( ) Yes ( ) No / When?
c Which foods should be introduced first when starting complementary feeding. ( ) Yes ( ) No / Which ones?
2. Did you receive prenatal care somewhere else than CESSA or medical unit? ( ) Yes ( ) No
If yes,
2.1 Where?
2.2 From whom?
2.3 Did you receive information about breastfeeding? ( ) Yes ( ) No
2.4 Did you receive information about complementary feeding? ( ) Yes ( ) No
3. From what sources, other than CESSA or medical unit, did you receive information about breastfeeding?
( ) Yes ( ) No Radio
( ) Yes ( ) No Television
( ) Yes ( ) No Magazine
( ) Yes ( ) No Newspaper
( ) Yes ( ) No Other: ____
4. During your prenatal care at the medical unit or hospital, were you given any of the following?
( ) Yes ( ) No Formula milk
( ) Yes ( ) No Bottles
( ) Yes ( ) No Pacifiers
( ) Yes ( ) No Nipple shield or any device to help with breastfeeding
( ) Yes ( ) No Other items promoting any of the above
4.1 Where did you receive these?

Section E: CURRENT BREASTFEEDING EXPERIENCE AND COMPLEMENTARY FEEDING PRACTICES
1. After hospital discharge, were you breastfeeding your baby exclusively?
( ) Yes ( ) No ( ) Don't know
2. Since your last baby was born, how many times have you returned to CESSA or medical unit?
2.1 Why did you return?

2.2 During those visits to CESSA or medical unit, have you received support for breastfeeding or complementary feeding? ( ) Yes ( ) No
3. Since your baby was born, have you had any disease that prevented you from breastfeeding your baby? ( ) Yes ( ) No
3.1 Which one?
3.2 How did it affect you?
If you stopped breastfeeding then,
3.3 Did you resume breastfeeding? ( ) Yes ( ) No
4. Since your baby was born, have you seeked medical care for your baby anywhere other than CESSA or medical unit? ( ) Yes ( ) No
If yes,
4.1 Where?
4.1 Why?
4.2 Who attended you?
4.3 Did you receive support for breastfeeding or complementary feeding? ( ) Yes ( ) No
5. Are you still breastfeeding your baby? ( ) Yes ( ) No
If yes,
5.1 Do you breastfeed exclusively, that is, without giving formula milk, water, tea, juice, cereals or other food? (Note: in EBF, medicine and rehydration serum are allowed)
If yes, confirm,
5.2 So, you have never given your baby formula milk, water, tea, etc.? ( ) Yes ( ) No
If no,
If still breastfeeding but NOT exclusively,
5.3. How old was your baby when you stopped breastfeeding her/him exclusively (that is, without giving formula milk, water, tea, juice, cereals or other food? Months / Weeks
If NOT breastfeeding any more,
5.4 How old was your baby when you stopped breastfeeding her/him completely? Months / Weeks
6. During your current breastfeeding experience, do/did you have discomfort in your breast or nipples? ( ) Yes ( ) No
If yes,
6.1 What kind of discomfort?

If the woman no longer breastfeeds or not exclusively:
7. Why did you start giving your baby food other than breast milk?
8. What other liquids do you give your baby?

Liquid	Yes / No	Baby's age when introduced
Formula milk		
Water		
Juice		
Tea		
Pozol		
Oatmeal with cocoa		
Atole		
Other		

9. Besides liquids, are you giving solid foods?
If yes,
9.1 Which ones?

Food	Yes / No	Baby's age when introduced
Vegetables		
Fruits		
Cereals, tubers		
Legumes		
Meat (including liver, viscera)		
Egg		
Dairy products		
Other		
